# 

**Published:** 1877-12

**Authors:** 


### CONTENTS:

ORIGINAL COMMUNICATIONS:
Affections of the Nipple and Breast, incident to early lactation. By Edw. Warren Sawyer, M.D....... 561
Intra-capsular Fracture of the Neck of the Femur. Recovery. By H. Wardner, M.D.................... 582
The Plaster of Paris Jacket in Spinal Diseases. By Edmund Andrews, A.M., M.D......................... 586
Case of Spontaneous Gangrene. By Abner Hard, M.D................. 592
Case of Twisted Pedicle. Ovariotomy. By K. Stansbury Sutton, A.M., M.D......................... 595
CLINICAL REPORTS:
St. Luke's Hospital. Service of Jno. E. Owens, M.D..................... 599
Notes from Private Practice........ 605
REVIEWS AND BOOK NOTICES.......... 608
EDITORIAL.................................. 625
CORRESPONDENCE.
Proposed Plan for Medical Reform. 626
"How to Maintain Discipline."..... 629
The Nuisance Created by Rendering Tanks and Manufacture of Fertilizers............................ 636
BOOKS AND PAMPHLETS RECEIVED..... 644
SUMMARY:
I. Surgery........................... 646
II. Therapeutics...................... 657
MEDICAL NEWS AND ITEMS. ........... 659
List of Licentiates................ 668
ANNOUNCEMENTS FOR THE MONTH.. ... 672

